# Supplementary material for: TET3 prevents terminal differentiation of adult NSCs by a non-catalytic action at Snrpn
Source: Nat Commun. 2019 Apr 12;10:1726. doi: 10.1038/s41467-019-09665-1 (PMC6461695; doi:10.1038/s41467-019-09665-1)
Supplement: Supplementary file 1 — Supplementary Information [file 41467_2019_9665_MOESM1_ESM.pdf]

Supplementary Information

TET3 prevents terminal differentiation of adult NSCs by a non-catalytic action at *Snrpn*

Montalbán-Loro et al.,

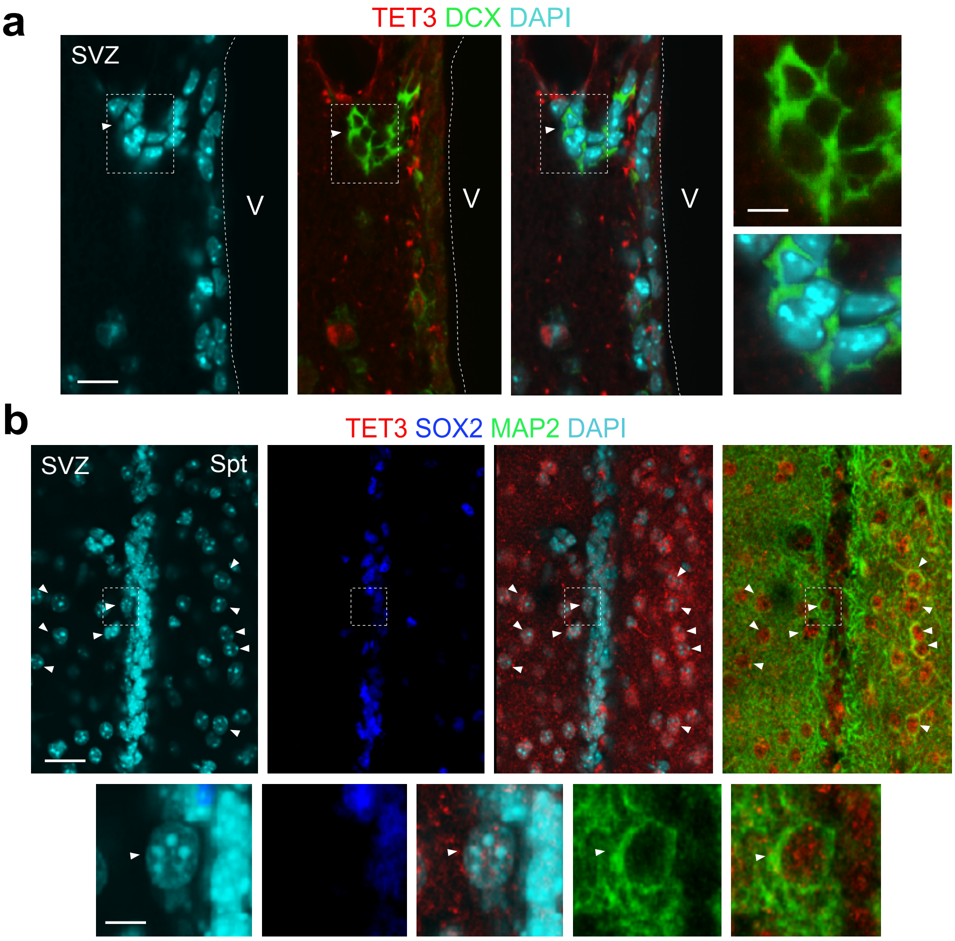

Supplementary Figure 1. TET3 is present in mature neurons but not in the neuroblast population. (a) Immunohistochemistry images for TET3 (red) and DCX (green) in the SVZ of adult wild-type mice. Dark arrowheads indicate DCX+ neuroblast cells. **(b)** Immunohistochemistry for TET3 (red), SOX2 (blue) and MAP2 (green) in the SVZ of adult wild-type mice. Dark arrowheads indicate MAP2+ neurons. High magnification images are shown. V: lateral ventricle lumen. DAPI was used to counterstain DNA. Scale bar in a and b: 20 μm (inserts, 8 μm).

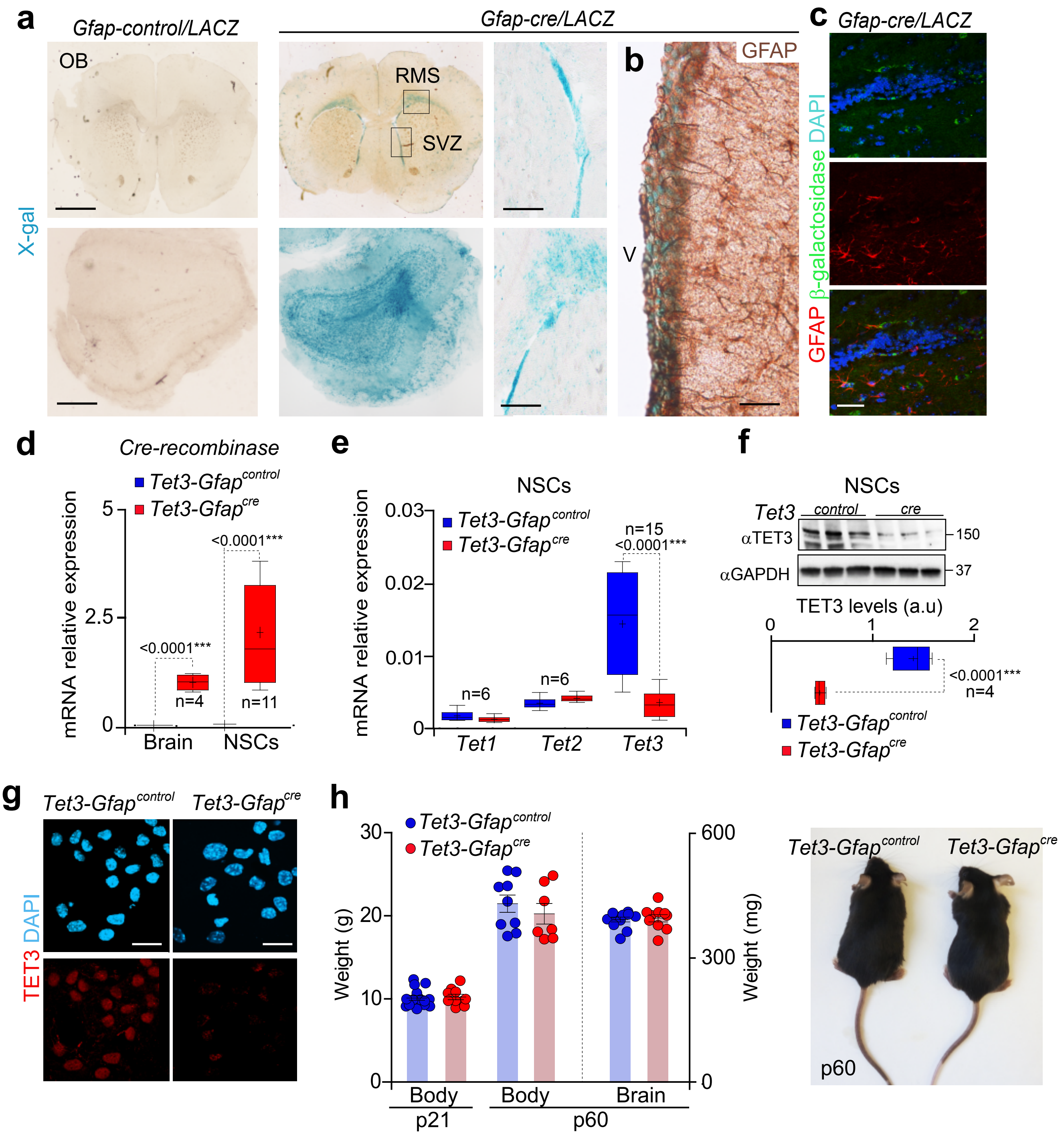

Supplementary Figure 2. Conditional deletion of *Tet3* occurs specifically in the GFAP+ population. **(a)** β-galactosidase staining (blue) in the SVZ, olfactory bulb (OB) and rostral migratory stream (RMS) of *Gfap-cre/LacZ mice. Gfap-control/LacZ* samples with no cre-recombinase were used as controls for the staining. **(b)** β-galactosidase staining (blue) and immunohistochemistry for GFAP in the SVZ of *Gfap-cre/LacZ* mice. **(c)** Immunohistochemistry images for β-galactosidase (green) and GFAP (red) in the SVZ of *Gfap-cre/LacZ* mice. **(d)** Quantitative PCR (qPCR) for *Cre-recombinase* in the brain and NSCs of *Tet3-Gfap^{control}* and *Tet3-Gfap^{cre}* mice. **(e)** qPCR for *Tet1, Tet2* and *Tet3* in *Tet3-Gfap^{control}* and *Tet3-Gfap^{cre}* NSCs. **(f)** Western-blot (WB) for TET3 in *Tet3-Gfap^{control}* and *Tet3-Gfap^{cre}* neurospheres cultures growing in proliferating conditions (upper panel). Quantification of TET3 protein levels by WB (lower panel). **(g)** Immunocytochemistry for TET3 (red) in proliferating neurospheres isolated from *Tet3-Gfap^{control}* and *Tet3-Gfap^{cre}* SVZ. DNA was counterstained with DAPI. **(h)** Body and brain weights in postnatal day (p) 21 and 60 mice from the two genotypes (left panel). Images of *Tet3-Gfap^{control}* and *Tet3-Gfap^{cre}* mice (right panel). V: lateral ventricle. Data are expressed relative to *Gapdh*. All error bars show s.e.m. P-values and number of samples are indicated. Scale bar in a, 1 mm (inserts in a, 100 μm); in b and c, 20 μm; in g, 10 μm.

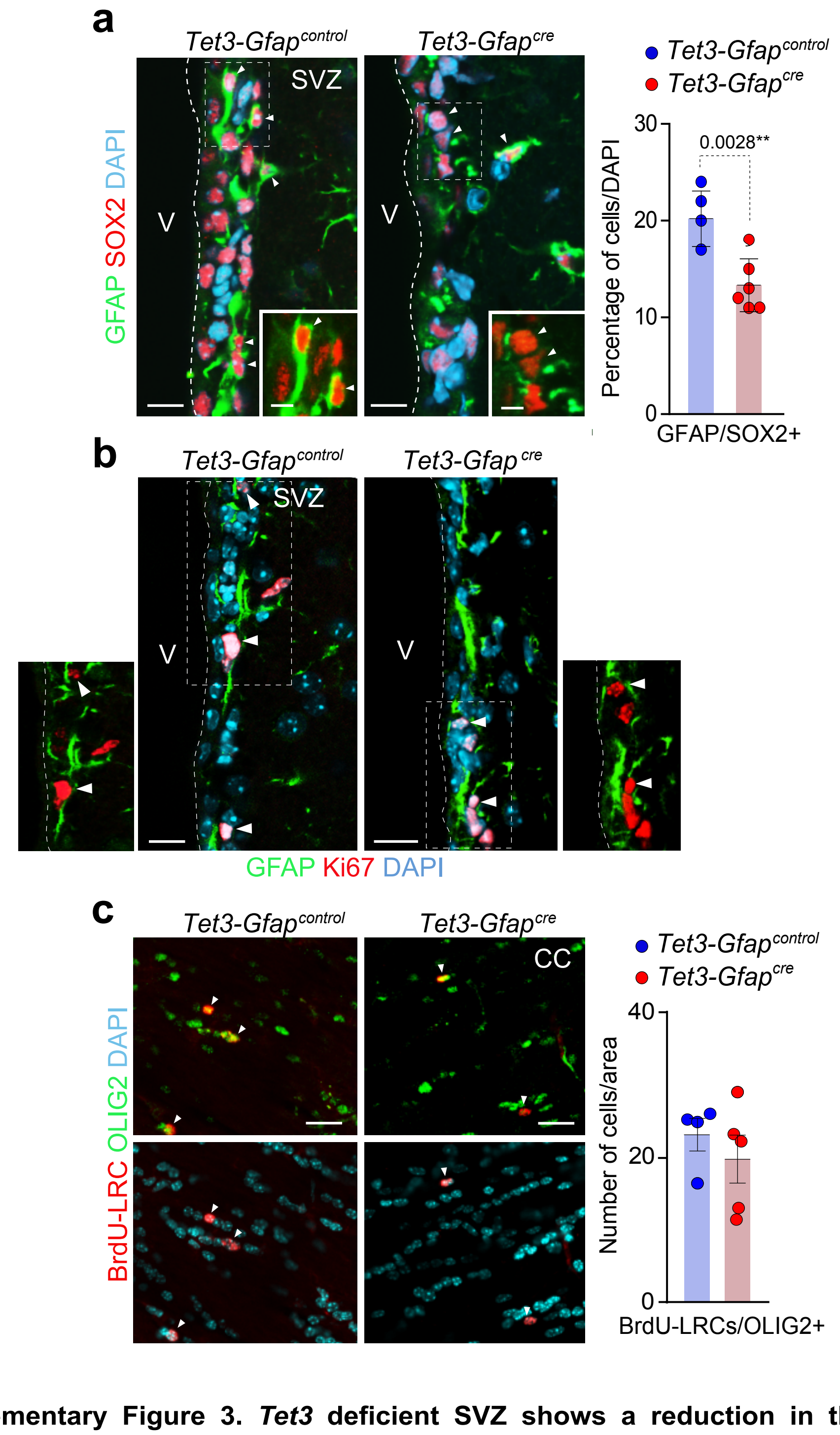

Supplementary Figure 3. *Tet3* deficient SVZ shows a reduction in the GFAP/SOX2+ population. (a) Immunohistochemistry images for the astrocyte marker GFAP (green) and the stem cell marker SOX2 (red) in the SVZ of *Tet3-Gfap^{control}* and *Tet3-Gfap^{cre}* mice (left panels). Percentage of GFAP/SOX2+ cells in the SVZ of *Tet3-Gfap^{control}* and *Tet3-Gfap^{cre}* mice (right panel). **(b)** Immunohistochemistry images for the proliferation marker ki67 (red) and GFAP (green) in the SVZ of *Tet3-Gfap^{control}* and *Tet3-Gfap^{cre}* mice. Dark arrowheads indicate GFAP/Ki67+ astrocyte cells. **(c)** Immunohistochemistry images for BrdU-LRC (red) and the oligodendrocyte marker OLIG2 (green) in the *corpus callosum* (CC) of *Tet3-Gfap^{control}* and *Tet3-Gfap^{cre}* mice. DAPI was used to counterstain DNA. Error bars show s.e.m. P-values and number of samples are indicated. V: ventricle lumen. Scale bar in a and b, 20 µm; in c, 30 µm.

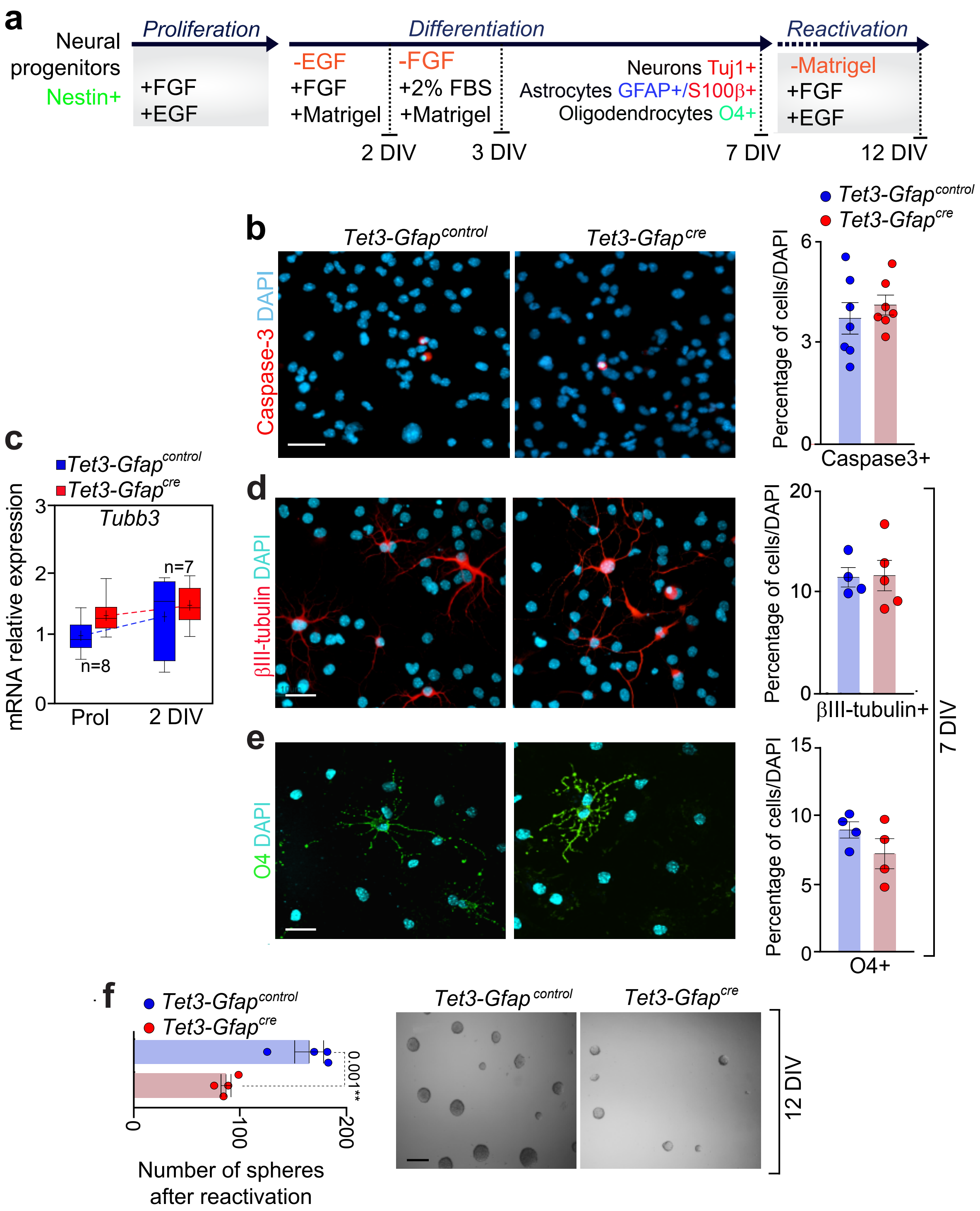

Supplementary Figure 4. Differentiated *Tet3-Gfap*^{cre} NSCs form less neurospheres after reactivation. (a) Schematic representation of the differentiation protocol. To induce differentiation, EGF was removed and cells were plated in matrigel for attachment. Two days after (2 DIV), FGF was removed and 10% FBS was added to the growth medium. Seven days after (7 DIV) cells were fixed for immunostaining. For the reactivation assay, cultures were tripsinized and cultured again in the presence of EGF and FGF and in the absence of matrigel. **(b)** Immunocytochemistry images for the activated Caspase-3 (red) in *Tet3-Gfap*^{control} and *Tet3-Gfap*^{cre} cultures (left panels). Percentage of Caspase-3+ cells in *Tet3-Gfap*^{control} and *Tet3-Gfap*^{cre} cultures (right panel). **(c)** qPCR for the neuronal marker *Tubb3* in *Tet3-Gfap*^{control} and *Tet3-Gfap*^{cre} NSCs in proliferation conditions and after 2 DIV of differentiation. Data are expressed relative to *Gapdh.* **(d)** Immunocytochemistry images for βIII-tubulin+ neurons in *Tet3-Gfap*^{control} and *Tet3-Gfap*^{cre} NSCs after 7 DIV of differentiation (left panels). Percentage of cells that are positive for βIII-tubulin in the two genotypes (right panel). **(e)** Immunocytochemistry images for O4+ oligodendrocytes in *Tet3-Gfap*^{control} and *Tet3-Gfap*^{cre} NSCs after 7 DIV of differentiation (left panels). Percentage of cells that are O4+ in the two genotypes (right panel). **(f)** Number of neurospheres formed after detaching and replating differentiated NSCs in proliferation conditions (left panel). Representative images of neurospheres formed from both genotypes after reactivation (right panels). DAPI was used to counterstain DNA. All error bars show s.e.m. P-values and number of samples are indicated. Scale bars in b, d and e, 40 μm; in f, 100 μm.

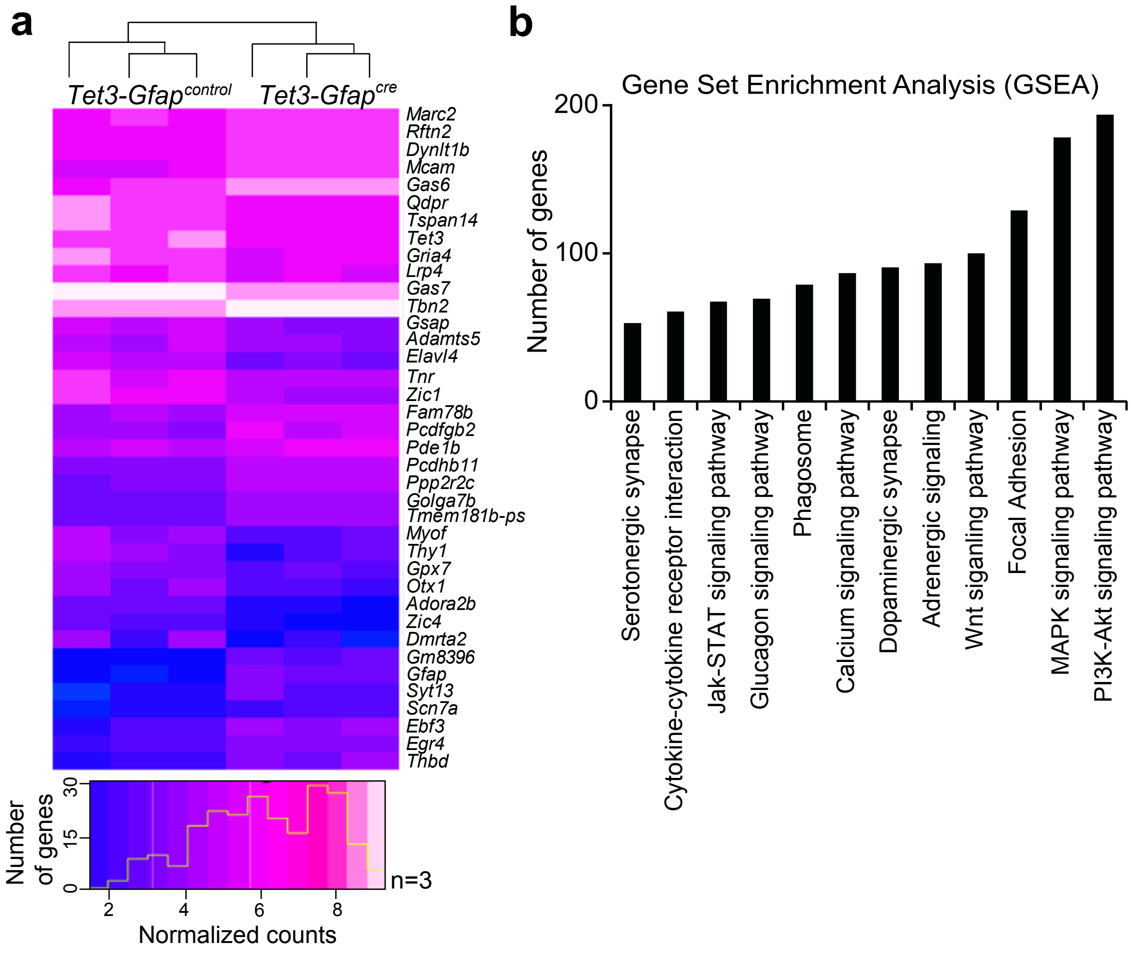

Supplementary Figure 5. RNAseq data show that depletion of *Tet3* causes significant changes in gene expression. (a) Hierarchical clustering and heatmap of RNAseq data showing expression of the top 20% of variable genes (FDR<1e-4) in *Tet3-Gfap*^{control} and *Tet3-Gfap*^{cre} NSCs. Different colours represent the normalized read counts value for each sample. Yellow line in the colour leyend indicates the number of genes for each value. **(b)** Gene Set Enrichment Analysis (GSEA) based on RNAseq data that shows statistically significant changes in sets of genes related to different pathways. Three samples of each genotype were used for the RNAseq analysis.

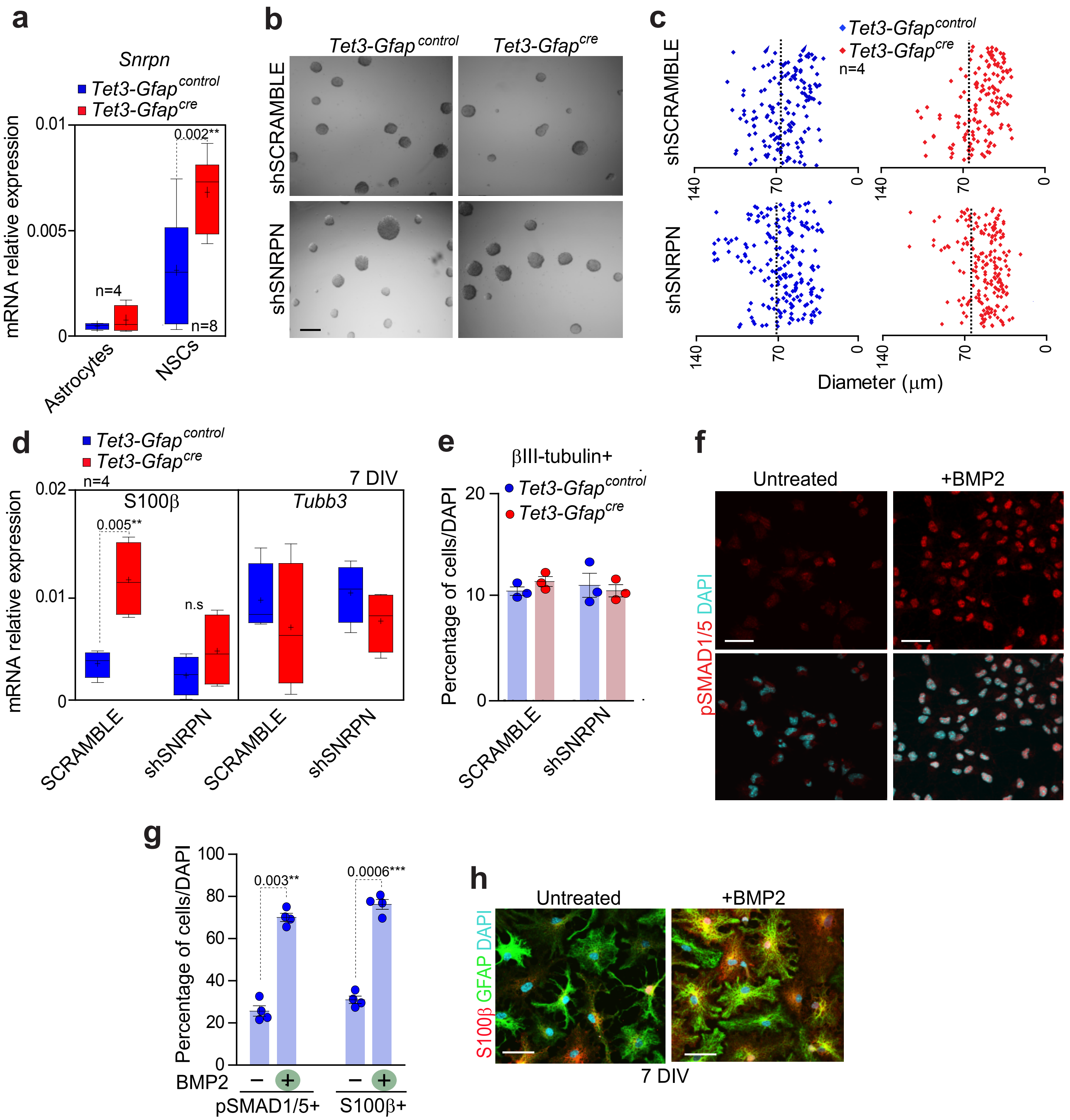

Supplementary Figure 6. Interference of *Snrpn* expression rescues self-renewal and differentiation defects in *Tet3-Gfap*^{cre} NSCs. (a) qPCR for *Tet3* in primary astrocytes and NSCs isolated from *Tet3-Gfap*^{control} and *Tet3-Gfap*^{cre} adult SVZ. Overexpression of *Snrpn* was seen in NSCs but not in astrocytes derived from *Tet3* deficient SVZ. **(b)** Phase contrast images of neurospheres obtained after shRNA for SNRPN in *Tet3-Gfap*^{control} and *Tet3-Gfap*^{cre} NSCs. shCRAMBLE was used as a control of gene interference. **(c)** Diameter of secondary spheres in shSCRAMBLE and shSNRPN conditions for both genotypes. Dashed lines represent the mean diameter for each condition. **(d)** qPCR for *S100β* and *Tubb3* in shSCRAMBLE and shSNRPN conditions in *Tet3-Gfap*^{control} and *Tet3-Gfap*^{cre} NSCs after 7 DIV of differentiation. **(e)** Percentage of positive cells for βIII-tubulin after 7 DIV of differentiation in shSCRAMBLE and shSNRPN conditions in both genotypes. **(f)** Immunocytochemistry images for pSMAD1/5 in wild-type NSCs treated or not with BMP2. **(g)** Percentage of positive cells for pSMAD1/5 and S100β after 7 DIV of differentiation in wild-type NSCs treated or not with BMP2. **(h)** Immunocytochemistry images for S100β (red) and GFAP (green) in wild-type NSCs treated or not with BMP2. Data are expressed relative to *Gapdh.* DNA was counterstained with DAPI. All error bars show s.e.m. P-values and number of samples are indicated. Scale bar in b, 100 μm; in f and h, 30 μm. .

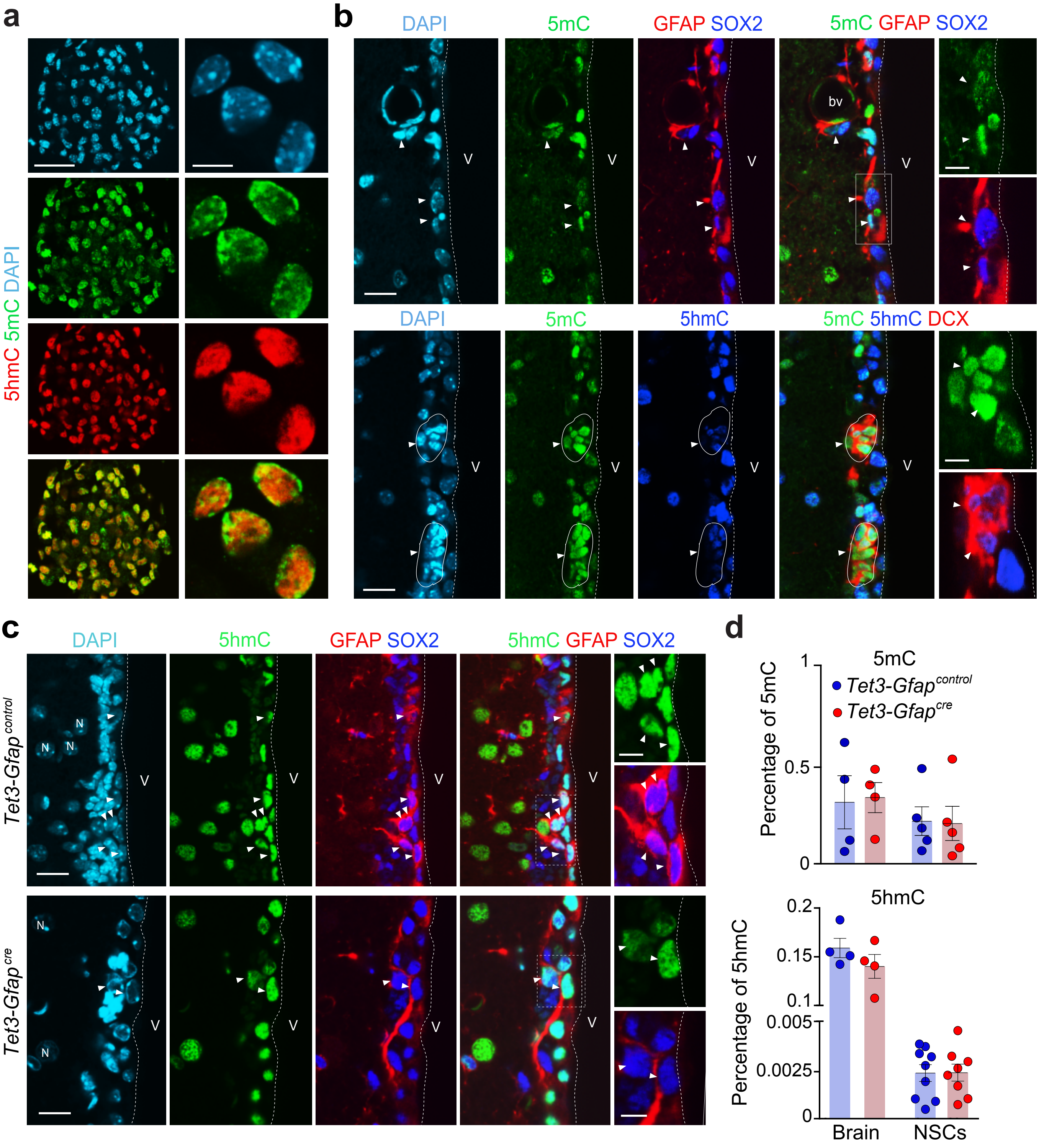

Supplementary Figure 7. Methylation and hydroxymethylation levels are maintained in *Tet3* deficient NSCs. (a) Immunocytochemistry for 5mC (green) and 5hmC (red) in neurospheres isolated and expanded *in vitro* from the adult wild-type SVZ. **(b)** Immunohistochemistry for 5mC (green) in double GFAP (red) and SOX2 (blue) positive cells within the SVZ of wild-type adult mice (upper panels). Immunohistochemistry for 5mC (green) and 5hmC (blue) in DCX (red) positive neuroblasts in the SVZ of wild-type mice (lower panels). **(c)** Immunohistochemistry for 5hmC (green) in GFAP (red) and SOX2 (blue) positive cells within the SVZ of *Tet3-Gfap*^{control} and *Tet3-Gfap*^{cre} mice. 5hmC is present in GFAP/SOX2+ cells close to the lateral ventricle wall. Dark arrowheads indicate GFAP/SOX2+ astrocytes cells with significant levels of 5hmC. DAPI was used to counterstain DNA. **(d)** Global percentage of 5mC and 5hmC in *Tet3-Gfap*^{control} and *Tet3-Gfap*^{cre} NSCs and brain. All error bars show s.e.m. Number of samples are indicated. Scale bars in a, left panels: 25 μm; right panels: 7 μm; In b and c: 20 μm (inserts in b and c, 8 μm).

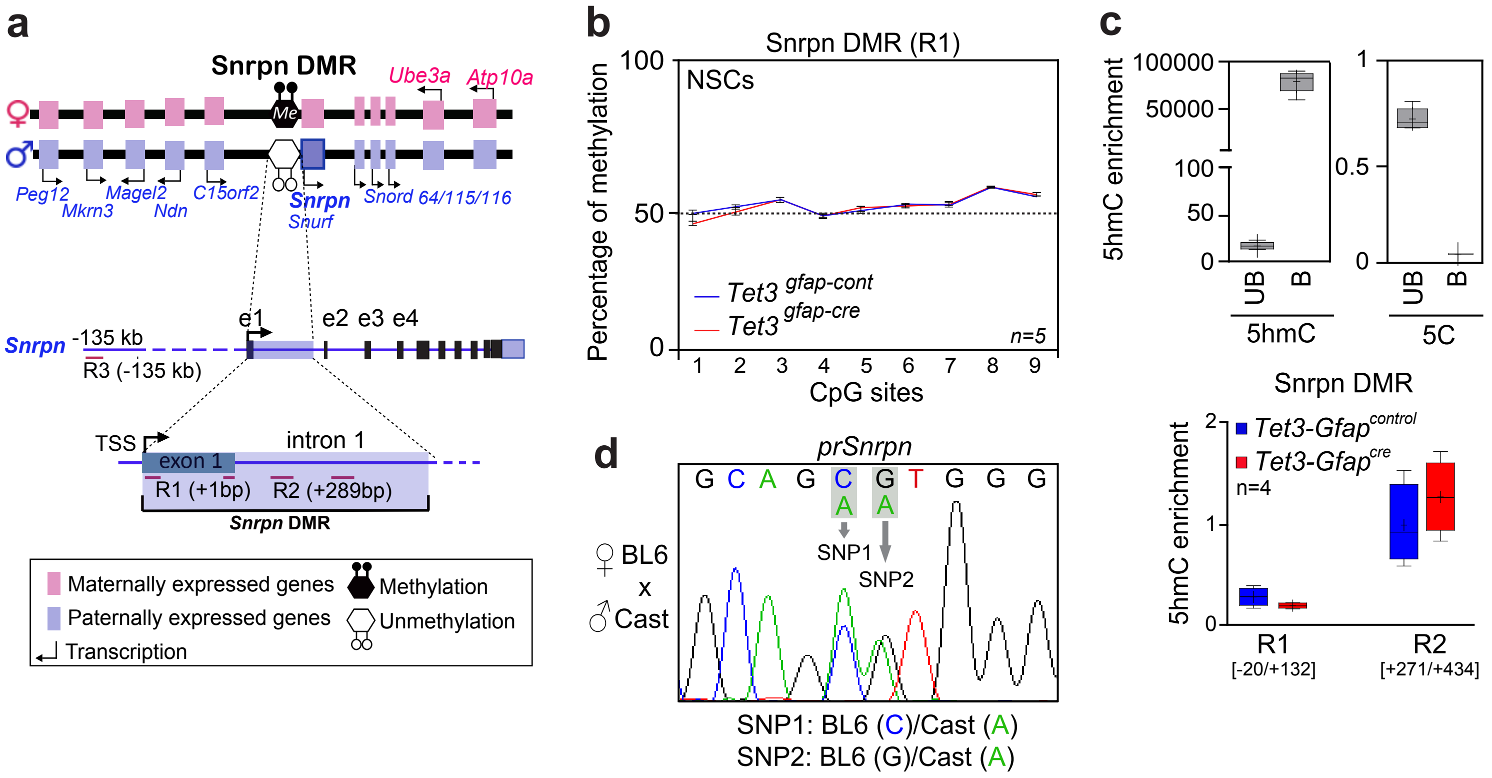

Supplementary Figure 8. Methylation at the *Snrpn* DMR is maintained in *Tet3* deficient NSCs. (a) Schematic of the *Snrpn* imprinted cluster. Expression of the maternally (pink) and paternally (blue) expressed genes within the cluster are shown. The methylation status of the *Snrpn* DMR is illustrated with open and filled circles representing full methylation at the maternal allele and lack of metylation at the paternal allele, respectively (upper panel). Schematic diagram showing the *Snrpn* gene and its DMR. Primers used are in red and numbered relative to the transcription start site (TSS). **(b)** Percentage of methylation determined by bisulfite conversion and pyrosequencing at several CpG sites within the *Snrpn* DMR in *Tet3-Gfap*^{control} and *Tet3-Gfap*^{cre} NSCs. **(c)** Controls for the enrichment of 5hmC in genomic DNA spiked with synthetic *Arabidopsis Thaliana* DNA, containing either 5hmC or 5C (upper panel). Values are shown as the fold change over the IgG. UB: unbound; B: bound. Enrichment of 5hmC in two different regions at the Snrpn DMR (R1 and R2) in *Tet3-Gfap*^{control} and *Tet3-Gfap*^{cre} NSCs (lower panel). **(d)** Genomic DNA sequence in F1 hybrid NSCs derived from *Mus musculus domesticus* (abbreviated, BL6) and *Mus musculus castaneus* (abbreviated, Cast) mice, showing two diagnostic strain-specific polymorphisms at the *Snrpn* promoter. SNP: single nucleotide polymorphism. All error bars show s.e.m. Number of samples are indicated.

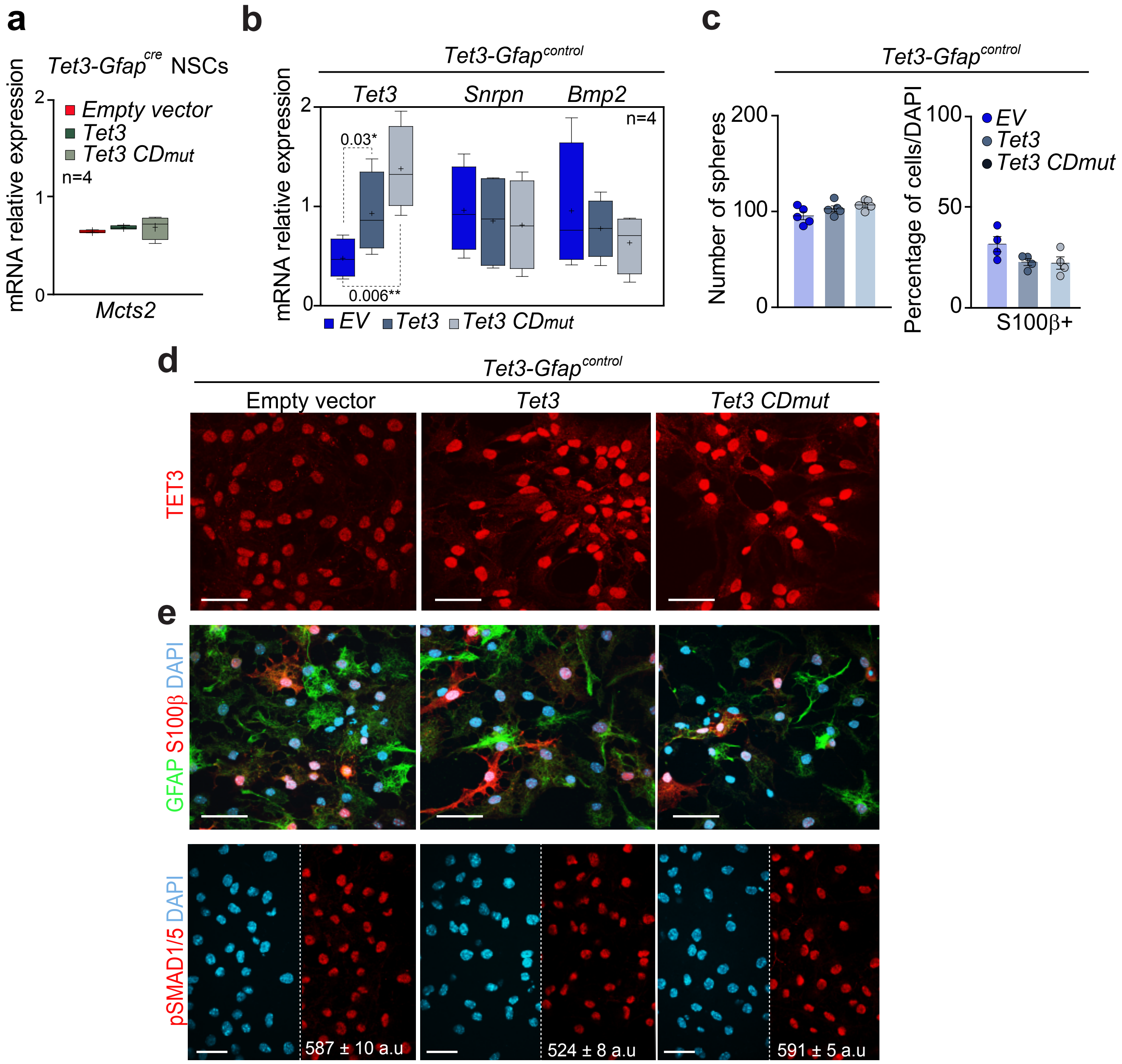

Supplementary Figure 9. Overexpression of *Tet3* does not modify self-renewal or differentiation capacities in wild-type NSCs. (a) qPCR for the imprinted gene *Mcts2* in *Tet3-Gfap^{cre}* NSCs that had been nucleofected with *Tet3* or with *Tet3* with a mutated catalytic domain (Tet3 CDmut). An empty vector was used as a control for nucleofection (EV). **(b)** qPCR for *Tet3, Snrpn* and *Bmp2* in *Tet3-Gfap^{control}* NSCs that had been nucleofected with *Tet3*, Tet3 CDmut or EV. **(c)** Number of spheres (left panel) and percentage of S100β+ cells (right panel) in *Tet3-Gfap^{control}* NSCs that had been nucleofected with *Tet3*, Tet3 CDmut or EV. **(d)** Immunocytochemistry images for TET3 (red) in *Tet3-Gfap^{control}* NSCs that had been nucleofected with *Tet3*, Tet3 CDmut or EV. **(e)** Immunocytochemistry images for S100β+ (red) and GFAP (green) in *Tet3-Gfap^{control}* NSCs that had been nucleofected with *Tet3*, Tet3 CDmut or EV (upper panels). Immunocytochemistry images for pSMAD1/5+ (red) in *Tet3-Gfap^{control}* NSCs that had been nucleofected with *Tet3*, *Tet3 CDmut* or EV (lower panels). Data are expressed relative to *Gapdh.* DAPI was used to counterstain DNA. All error bars show s.e.m. P-values and number of samples are indicated. Scale bars in d and e, 30 μm.

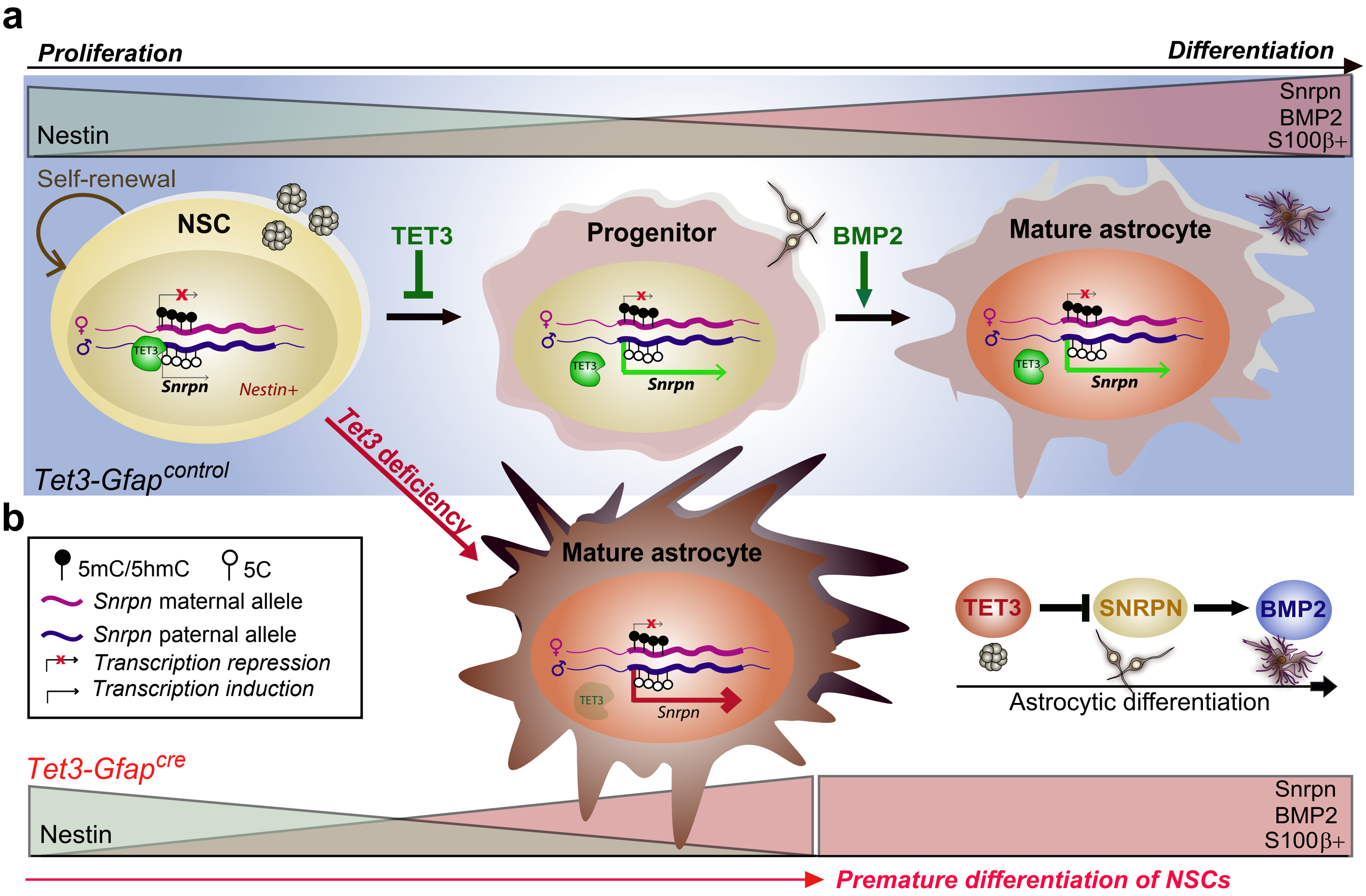

Supplementary Figure 10. TET3 binds to the paternal allele at the *Snrpn* promoter to repress its expression and to prevent BMP-mediated terminal differentiation of NSCs. (a) Schematic drawing of the process of adult NSCs differentiation into mature astrocytes. Nestin+ NSCs proliferate and self-renew in the presence of mitogen stimulation. During proliferation TET3 is bound to the *Snrpn* promoter. The *Snrpn* gene is an imprinted gene expressed from the paternal unmethylatrd allele and silent from the maternal methylated allele. TET3 binds to the paternal allele repressing *Snrpn* expression and contributing to the maintenance of NSCs in an undifferentiated state. SNRPN drives *Bmp2* expression which induce terminal differentiation of multipotent NSCs into non-neurogenic S100β+ astrocytes. **(b)** Loss of TET3 results in increased levels of SNRPN (and consequenctly of BMP2) early during differentiation of NSCs, which induces premature differentiation of multipotent NSCs into astrocytes that lack stem cell properties.

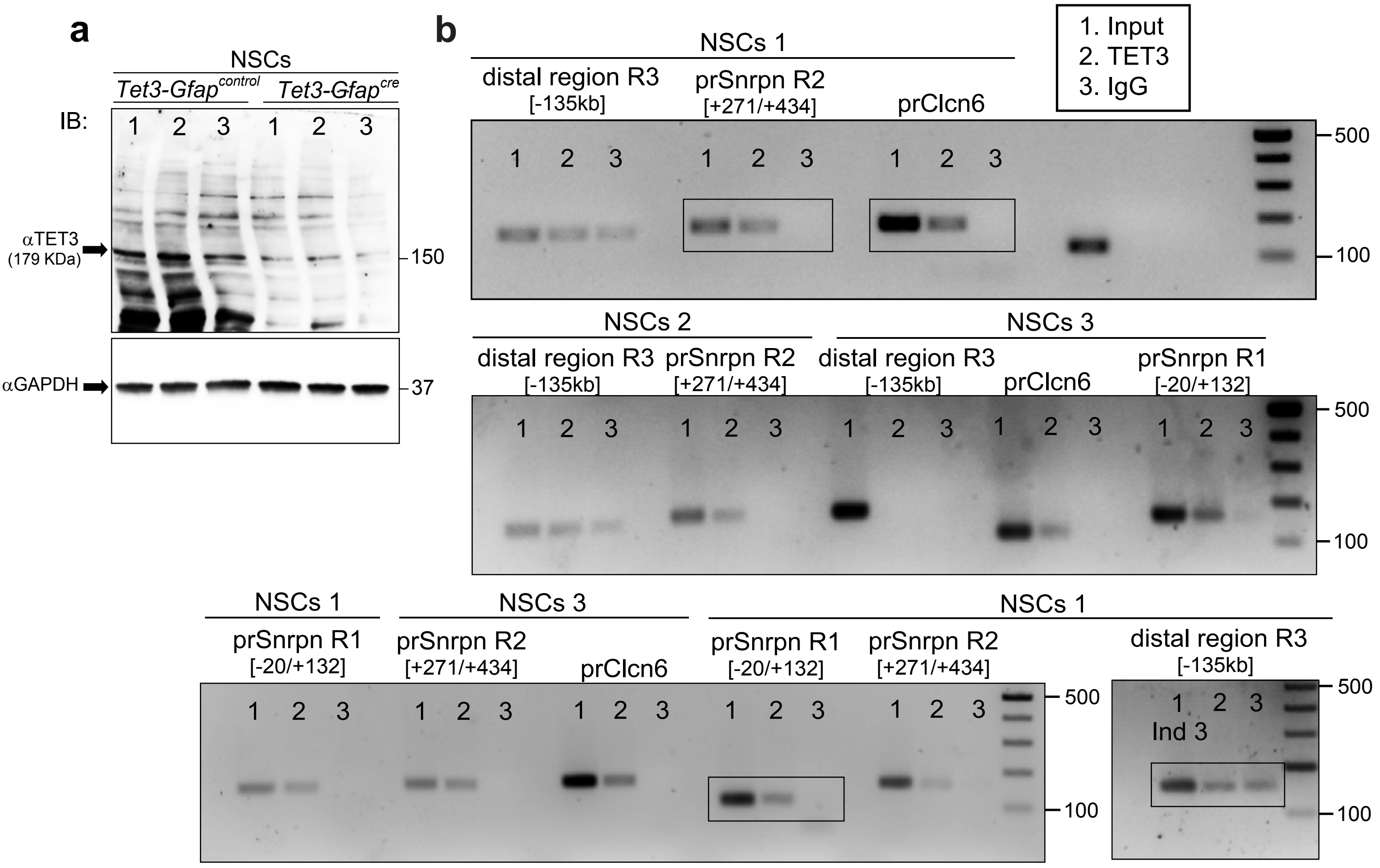

Supplementary Figure 11. Uncropped western blot (WB) and chromatin immunoprecipitation (ChIPs) qPCRs. **(a)** Immunoblots (IB) in Supplementary Figure 2f. Three independent neurosphere cultures from *Tet3-Gfap^control^* and *Tet3-Gfap^cre^* SVZ were used. **(b)** qPCRs after ChIP with TET3 antibody of wild-type NSCs shown in Figure 6a.

Supplementary Tables

Supplementary Table 1. List of Syber-green primers used.

Gene	Sequence (5'-3')	Application
Clcn6-F	CAACTCCTGAGGACCTGACA	ChIP
Clcn6-R	GCTCAGACACAGCCTCCTCT	ChIP
Cre-F	GCGGTCTGGCAGTAAAAACTATC	Genotyping/Expression analysis
Cre-R	GTGAAACAGCATTGCTGTCACTT	Genotyping/Expression analysis
Cre-F wt	CTAGGCCACAGAATTGAAAGATCT	Genotyping
Cre-R wt	GTAGGTGGAAATTCTAGCATCATCC	Genotyping
Gapdh-F	GAACATCATCCCTGCATCCA	Expression analysis
Gapdh-R	CCAGTGAGCTTCCCGTTC A	Expression analysis
SnrpnDMR-R1-F	GGCAAAAATGTGCGCATGTG	ChIP/Expression analysis
SnrpnDMR-R1-R	ATTGCGTTGCAAATCACTCC	ChIP/Expression analysis
SnrpnDMR-R2-F	ACTCCTTGGGTGTGTTAGTG	ChIP/ChIP in BxC
SnrpnDMR-R2-R	GACTTCCAGGAGTCCAGAGG	ChIP/ChIP in BXC
Snrpn-R3F	TATGGCCGCCTACTTTTGTC	ChIP
Snrpn-R3R	AGGACCTGCTGCACTGACTT	ChIP
Snrpn-DMR-F	TTGGTAGTTGTTTTTTGGTAGGAT	Bisulfite sequencing
Snrpn-DMR-R	TCCACAAACCCAACTAACCTTC	Bisulfite sequencing
Tet3-R	GTGCAGTTGCTCGTCCTCAG	Expression analysis
Tet3-F	AGACCCTTCTCAGGGGTCAC	Expression analysis
Tet3-F	TACCTCTGCCTCTGGAGTGCTAA	Genotyping
Tet3-R1	ATGGCTACTCACAACCCAGTGAC	Genotyping
Tet3-R2	GTCAGGAAAGTCACATGGTTGTTG	Genotyping

Supplementary Table 2. List of primary antibodies used.

Antibody	Source	Host	Dilution	Cat #	Application
5hmC	Active Motif	Rabbit	1/1000	39769	ICC/IHC/E
5hmC	Diagenode	Mouse	1/1000	C15200200-50	E
5mC	Diagenode	Mouse	1/1000	C15200006	ICC/IHC/E
BrdU	Abcam	Rat	1/500	ab6326	ICC/IHC
Caspase 3	Cell Signaling	Rabbit	1/300	9661	ICC
DCX	Santa Cruz	Goat	1/300	sc-8066	IHC
GAPDH	Millipore	Mouse	1/5000	MAB374	WB
GFAP	Dako	Rabbit	1/600	Z0334	IHC
GFAP	Millipore	Chicken	1/600	AB5541	ICC/IHC
Ki67	Abcam	Rabbit	1/100	ab15580	IHC
MAP2	Chemicon	Mouse	1/500	MAB3418	IHC
Nestin	Hybridoma Bank	Mouse	1/4	rat-401	ICC
O4	Hybridoma Bank	Mouse	1/2	rip	ICC
OLIG2	Millipore	rabbit	1:500	AB9610	IHC
PSA-NCAM	AbCys S.A.	Mouse	1/300	ABC0019	IHC
pSMAD1/5	Cell Signaling	Rabbit	1:200	9516	ICC
S100β	Dako	Rabbit	1/300	Z0311	ICC/IHC
SNRPN	Abcam	Rabbit	1/300	Ab22430	ICC
Sox2	R&D Systems	Goat	1/200	AF2018	ICC/IHC
TET3	Abnova	Rabbit	1/300	PAB25635	ICC/IHC
TET3	Santa Cruz	Rabbit	1/100	sc-139186	ICC/WB
TET3	Millipore	Rabbit	1/10	ABE290	ChIP
βIII-tubulin	Covance	Mouse	1/300	PRB-435P	ICC
β-catenin	Cell Signaling	Rabbit	1/300	9587	IHC
γ-tubulin	Santa Cruz	Goat	1/300	sc-7396	IHC

ICC, Immonocytochemistry IHC, Immunohistochemistry WB, Western-blot
E, ELISA ChIP, Chromatin Immnoprecipitation

Supplementary Table 3. List of secondary antibodies used.

Antibody	Source	Dilution	Cat #	Application
Alexa Fluor® 488 Donkey Anti-Chicken	Jackson ImmunoResearch	1/600	703-545-155	IHC
Alexa Fluor® 488 Donkey Anti-Goat	Jackson ImmunoResearch	1/600	705-545-003	ICC
Alexa Fluor® 488 Donkey Anti-Mouse	Molecular Probes	1/600	A-21202	ICC/IHC
Alexa Fluor® 488 Donkey Anti-Rabbit	Jackson ImmunoResearch	1/600	711-547-003	ICC/IHC
Alexa Fluor® 647 Donkey Anti-Chicken	Jackson ImmunoResearch	1/600	703-605-155	ICC/IHC
Alexa Fluor® 647 Donkey Anti-Rabbit	Jackson ImmunoResearch	1/600	711-607-003	IHC
Cy3-Donkey Anti-Rabbit	Jackson ImmunoResearch	1/800	711-165-152	ICC/IHC
Cy3-Donkey Anti-Mouse	Jackson ImmunoResearch	1/800	715-165-151	ICC/IHC
Cy3-Donkey Anti-Rat	Jackson ImmunoResearch	1/800	712-165-153	ICC
Cy3-Donkey Anti-Goat	Jackson ImmunoResearch	1/600	705-166-147	IHC
Goat Anti-Mouse IgG-HRP	Dako	1/5000<br>1/2000	P0447	WB<br>E
Goat Anti-Rabbit IgG-HRP	Santa Cruz	1/5000<br>1/2000	sc-2004	WB<br>E

ICC, Immunocytochemistry IHC, Immunohistochemistry WB, Western-blot E, ELISA

Supplementary Table 4. List of Taqman probes used.

Gene	TaqMan Code (Applied Biosystems)
Cntn3	Mm00500947_m1
Cobl	Mm01187905_m1
Gapdh	Mm99999915_g1
Nes	Mm00450205_m1
S100β	Mm00485897_m1
Snrpn	Mm04204818_m1
Tet1	Mm01169087_m1
Tet2	Mm00524395_m1
Tet3	Mm00805756_m1
Tubb3	Mm00727586_s1
Mcts2	Mm00481540_s1
Bmp2	Mm01340178_m1
