# Supplementary material for: TET3 prevents terminal differentiation of adult NSCs by a non-catalytic action at Snrpn
Source: Nat Commun. 2019 Apr 12;10:1726. doi: 10.1038/s41467-019-09665-1 (PMC6461695; doi:10.1038/s41467-019-09665-1)
Supplement: Supplementary file 3 — Reporting Summary [file 41467_2019_9665_MOESM3_ESM.pdf]

nature research

Corresponding author(s): NCOMMS-18-28439

Last updated by author(s): Feb 24, 2019

Reporting Summary

Nature Research wishes to improve the reproducibility of the work that we publish. This form provides structure for consistency and transparency in reporting. For further information on Nature Research policies, see Authors & Referees and the Editorial Policy Checklist.

Statistics

For all statistical analyses, confirm that the following items are present in the figure legend, table legend, main text, or Methods section.

n/a	Confirmed	
☐	☒	The exact sample size (*n*) for each experimental group/condition, given as a discrete number and unit of measurement
☐	☒	A statement on whether measurements were taken from distinct samples or whether the same sample was measured repeatedly
☐	☒	The statistical test(s) used AND whether they are one- or two-sided<br>*Only common tests should be described solely by name; describe more complex techniques in the Methods section.*
☒	☐	A description of all covariates tested
☐	☒	A description of any assumptions or corrections, such as tests of normality and adjustment for multiple comparisons
☐	☒	A full description of the statistical parameters including central tendency (e.g. means) or other basic estimates (e.g. regression coefficient) AND variation (e.g. standard deviation) or associated estimates of uncertainty (e.g. confidence intervals)
☐	☒	For null hypothesis testing, the test statistic (e.g. *F*, *t*, *r*) with confidence intervals, effect sizes, degrees of freedom and *P* value noted<br>*Give P values as exact values whenever suitable.*
☒	☐	For Bayesian analysis, information on the choice of priors and Markov chain Monte Carlo settings
☒	☐	For hierarchical and complex designs, identification of the appropriate level for tests and full reporting of outcomes
☒	☐	Estimates of effect sizes (e.g. Cohen's *d*, Pearson's *r*), indicating how they were calculated

Our web collection on statistics for biologists contains articles on many of the points above.

Software and code

Policy information about availability of computer code

Data collection	No software was used.
Data analysis	To analyze the data we have used SPSS, version 24.0.0 for windows, and licended by the University of Valencia.

For manuscripts utilizing custom algorithms or software that are central to the research but not yet described in published literature, software must be made available to editors/reviewers. We strongly encourage code deposition in a community repository (e.g. GitHub). See the Nature Research guidelines for submitting code & software for further information.

Data

Policy information about availability of data

All manuscripts must include a data availability statement. This statement should provide the following information, where applicable:

- Accession codes, unique identifiers, or web links for publicly available datasets
- A list of figures that have associated raw data
- A description of any restrictions on data availability

Source data for figures are provided with the paper as "Source Data". The authors declare that all other data supporting the findings of this study are available within the paper and its supplementary information files. RNAseq raw data are available in GeneBank and accession code is included in Data availability statement.

Field-specific reporting

Please select the one below that is the best fit for your research. If you are not sure, read the appropriate sections before making your selection.

☒ Life sciences ☐ Behavioural & social sciences ☐ Ecological, evolutionary & environmental sciences

For a reference copy of the document with all sections, see nature.com/documents/nr-reporting-summary-flat.pdf

Life sciences study design

All studies must disclose on these points even when the disclosure is negative.

Sample size	No samples size was calculated previous the study. We analyzed a number of samples that together fullfill gaussian distribution.
Data exclusions	No data were excluded.
Replication	Culture experiments were done at least three independent times . In vivo experiments were done at least in two independent experiments
Randomization	Samples and mice were allocated randomly into experimental groups
Blinding	Blinding was used for all experiments.

Reporting for specific materials, systems and methods

We require information from authors about some types of materials, experimental systems and methods used in many studies. Here, indicate whether each material, system or method listed is relevant to your study. If you are not sure if a list item applies to your research, read the appropriate section before selecting a response.

Materials & experimental systems

n/a	Involved in the study	
☐	☒	Antibodies
☐	☒	Eukaryotic cell lines
☒	☐	Palaeontology
☐	☒	Animals and other organisms
☒	☐	Human research participants
☒	☐	Clinical data

Methods

n/a	Involved in the study	
☒	☐	ChIP-seq
☐	☒	Flow cytometry
☒	☐	MRI-based neuroimaging

Antibodies

Antibodies used	This information is located at Supplementary Table S1a. We have indicated Antibody Source, Host, Dilution used, catalogue number and application.
Validation	TET3 and SNRPN antibodies were validated in deficient NSCs. TET3 antobodies were additionally validated in TET3 overexpression experiments. Secondary antibodies controls were also used.

Eukaryotic cell lines

Policy information about cell lines

Cell line source(s)	Primary cultures of Neural Stem cells isolated from the Subventricular zone of adult mice and expanded in vitro.
Authentication	None of the cell lines used were authenticated
Mycoplasma contamination	All cell lines were negative for Mycoplasm contamination. Cell lines were tested by PCR with specific primers.
Commonly misidentified lines (See ICLAC register)	n/a

Animals and other organisms

Policy information about studies involving animals; ARRIVE guidelines recommended for reporting animal research

Laboratory animals	Mice: mus musculus domesticus and mus musculus castaneous. We used males and females from 2 to 4-months of age.
Wild animals	The study did not involve wild animals
Field-collected samples	The study did not involve samples collected from the field
Ethics oversight	Housing and experiments were carried out following protocols approved by the ethics committee of the University of Valencia (Spain).

Note that full information on the approval of the study protocol must also be provided in the manuscript.

Flow Cytometry

Plots

Confirm that:

☒ The axis labels state the marker and fluorochrome used (e.g. CD4-FITC).

☒ The axis scales are clearly visible. Include numbers along axes only for bottom left plot of group (a 'group' is an analysis of identical markers).

☒ All plots are contour plots with outliers or pseudocolor plots.

☒ A numerical value for number of cells or percentage (with statistics) is provided.

Methodology

Sample preparation	Neural stem cells isolated from the adult subventricular zone were stained using the BD CycletestTM Plus DNA kit in accordance with the manufacture´s protocol.
Instrument	Cell cycle phases were assessed in a FACS Verse flow cytometer (BD).
Software	Cell cycle phases data were analyzed with FlowJo® software.
Cell population abundance	For the cell cycle analysis at least 20 thousands events were analyzed. Approximately 90% of the population was considered for cell cycle based on SSC and FSC.
Gating strategy	Non-stained NSCs were used to establish boundaries between negative and positive cells.

☒ Tick this box to confirm that a figure exemplifying the gating strategy is provided in the Supplementary Information.
