# Supplementary material for: TET3 prevents terminal differentiation of adult NSCs by a non-catalytic action at Snrpn
Source: Nat Commun. 2019 Apr 12;10:1726. doi: 10.1038/s41467-019-09665-1 (PMC6461695; doi:10.1038/s41467-019-09665-1)
Supplement: Supplementary file 4 — Description of Additional Supplementary Files [file 41467_2019_9665_MOESM4_ESM.pdf]

Description of Additional Supplementary Files

File Name: Supplementary Data 1
Description: corresponds to the list of significantly upregulated genes obtained from the RNAseq analysis.

File Name: Supplementary Data 2
Description: corresponds to the list of significantly downregulated genes obtained from the RNAseq analysis.

File Name: Supplementary Data 3
Description: corresponds to the list of significantly downregulated and upregulated imprinted genes.
